# Elevated inflammatory gene expression in intervertebral disc tissues in mice with ADAM8 inactivated

**DOI:** 10.1038/s41598-021-81495-y

**Published:** 2021-01-19

**Authors:** Yejia Zhang, Zuozhen Tian, David Gerard, Lutian Yao, Frances S. Shofer, Gabriella Cs-Szabo, Ling Qin, Maurizio Pacifici, Motomi Enomoto-Iwamoto

**Affiliations:** 1 Department of Physical Medicine & Rehabilitation, Perelman School of Medicine, University of Pennsylvania, Philadelphia, USA; 2 Department of Orthopedic Surgery, Perelman School of Medicine, University of Pennsylvania, Philadelphia, USA; 3 Emergency Medicine, Perelman School of Medicine, University of Pennsylvania, Philadelphia, USA; 4 Translational Musculoskeletal Research Center (TMRC), Corporal Michael J. Crescenz Veterans Affairs Medical Center, Philadelphia, PA USA; 5 Department of Biochemistry, Rush University Medical Center, Chicago, IL USA; 6 Department of Orthopaedics/Sports Medicine and Joint Surgery, First Affiliated Hospital, China Medical University, Shenyang, Liaoning China; 7 Division of Orthopaedic Surgery, The Children’s Hospital of Philadelphia, Philadelphia, USA; 8 Department of Orthopedics, University of Maryland, Baltimore, MD USA

**Keywords:** Cell biology, Diseases, Pathogenesis

## Abstract

We found ADAM8 enzymatic activity elevated in degenerative human intervertebral disc (IVD). Here, we examined the discs in ADAM8-inactivation mice that carry a mutation preventing self-activation of the enzyme. Surprisingly, elevated gene expression for inflammatory markers (*Cxcl1*, *IL6*) was observed in injured discs of ADAM8 mutant mice, along with elevated expression of type 2 collagen gene (*Col2a1*), compared with wild type controls. Injured annulus fibrosus of mutant and wild type mice contained a higher proportion of large collagen fibers compared with intact discs, as documented by microscopic examination under circular polarized light. In the intact IVDs, *Adam8*^*EQ*^ mouse AF contained lower proportion of yellow (intermediate) fiber than WT mice. This suggests that ADAM8 may regulate inflammation and collagen fiber assembly. The seemingly contradictory findings of elevated inflammatory markers in mutant mice and excessive ADAM8 activity in human degenerative discs suggest that ADAM8 may interact with other enzymatic and pro-inflammatory processes needed for tissue maintenance and repair. As a future therapeutic intervention to retard intervertebral disc degeneration, partial inhibition of ADAM8 proteolysis may be more desirable than complete inactivation of this enzyme.

## Introduction

Chronic low back pain, a common clinical problem, has been estimated to be discogenic in origin 40% of the time^1^. Oegema et al. have found increased amounts of fibronectin fragments in human intervertebral disc (IVD) tissues^2^. We have subsequently identified ADAM8 (A Disintegrin and metalloproteinase domain-containing protein 8) as a fibronectin-cleaving enzyme in human degenerative IVDs^3^. We have shown that activated ADAM8, along with its fibronectin cleavage products, increased with human IVD degeneration^3^. Furthermore, biologically active fibronectin fragments have been shown to alter IVD cell metabolism in vitro^4^, and accelerate disc degeneration in rabbits in vivo^5^. These findings firmly establish the clinical relevance of ADAM8 as a candidate for key initiator of IVD degeneration.

ADAM8, a membrane-anchored protein structurally related to snake venom disintegrins, was originally described in inflammatory processes^6,7^. ADAM family members have been increasingly recognized in tumorigenesis^8^ and respiratory tract allergic and inflammatory processes^9^. They modulate cytokines, growth factors and their receptors and adhesion molecules via their proteolytic activities^10,11^. The ADAM8 ectodomain contains a metalloproteinase domain, a disintegrin domain, and a cysteine-rich region. A transmembrane domain anchors the molecule to the membrane, and connects to the cytoplasmic domain which contains a src homology 2 domain. The *Adam8* gene is transcribed and encodes an inactive enzyme that self-activates^12^. One amino acid change [replacing the Glutamic acid (E) at position 330 with a Glutamine (Q)] prevents autocatalytic prodomain removal^13^. Substrates of activated ADAM8 overlap with those of ADAM10 and 17, both major shedding enzymes which cleave proteins with immune functions and cell adhesion proteins^14^. Examples of ADAM8 substrates with immune function include the chemokine (C-X-C motif) ligand-1 (CXCL1)^14^, which has been included as a reporter gene in the current study. The choice of this marker is of particular clinical relevance, because IL8 (the human homologue of mouse CXCL1) was found in human IVD tissues. IL8 production and gene expression were elevated many fold post inflammatory cytokine stimulation^15^. Furthermore, *Cxcl1* gene expression was induced by injury in the mouse IVD^16,17^. Therefore, CXCL1, was used as a surrogate for disc injury repair/inflammation. ADAM8 has been shown to cleave fibronectin at theVRAA271↓272VYQP scissile bond^271^ in articular cartilage^18,19^ and IVD^3^. Considering the similarities between IVD and articular cartilage, and that fibronectin fragments stimulate extracellular matrix catabolic activity in IVDs^4,5^, we hypothesized that inhibition of ADAM8 enzymatic activity retards disc degeneration.

*Adam8*-deletion (lacking the entire molecule) mice show only minor developmental defects^20,21^, and increased neovascularization in an oxygen-induced retinopathy model^22^. Furthermore, ADAM8-deletion mice are highly resistant to development of experimental asthma^23^. To focus on the role of ADAM8 proteolytic function, a novel ADAM8-inactivation mouse model has been generated by introducing a point mutation, replacing the Glutamic acid at position 330 with a Glutamine (*Adam8*^*E330Q/E330Q*^; abbreviated as *Adam8*^*EQ*^)^24^. This mutated ADAM8 lacks its proteolytic activity, because the point mutation prevents ADAM8 activation by shedding its pro-domain^13^. These mice appear normal in development, with body weights comparable to those of wild type (WT) mice on the DBA background, while they exhibit reduction in incidence and severity of collagen-induced arthritis^24^.

We validated and improved^17,25^ the existing IVD injury models^26,27^. Our main contribution to improvement of the model is by using a transcutaneous approach, rather than cutting the overlying skin and ligaments. By using a transcutaneous approach, collateral damages and inflammation are limited, and the inflammatory markers better reflect those of IVD injury than those in the open approach. Consistent histological and molecular changes post disc injury have been shown at 1 week post injury^16,17,28^; therefore, this time point has been used in the current study to study an early phase of the repair process. The histological features are similar at 1 and 2 weeks post injury, and the disc healed with fibrocartilaginous tissue at the 4-week time point^17^. In this model, injury to tail IVD upregulates *Adam8* gene expression^17^, in mice on both C57BL/6 and DBA genetic backgrounds^16^, which is consistent with observations in human degenerative IVDs^3^. A limited set of molecular markers were selected as surrogates to reflect disc repair/inflammation post injury. IL8 (homologue of the mouse CXCL1), present in human IVD tissues, was elevated many fold post inflammatory cytokine stimulation^15^. Similarly, IL6 has been identified in human degenerative and herniated IVDs^29^. IL6 constitutes an emergency warning signal of tissue damage or infection, and its gene expression is tightly regulated both transcriptionally and posttranscriptionally^30^. Therefore, CXCL1 and IL6 have been used as surrogate markers for IVD inflammation. To examine whether inactivating ADAM8 protects the IVD from accelerated degeneration and inflammation, histological features and inflammatory markers in injured *Adam8*^*EQ*^ mouse tail IVDs were compared with those in WT mice.

## Results

### A point mutation in the ADAM8 proteolytic domain abrogates its ability to cleave fibronectin

**Figure 1:**
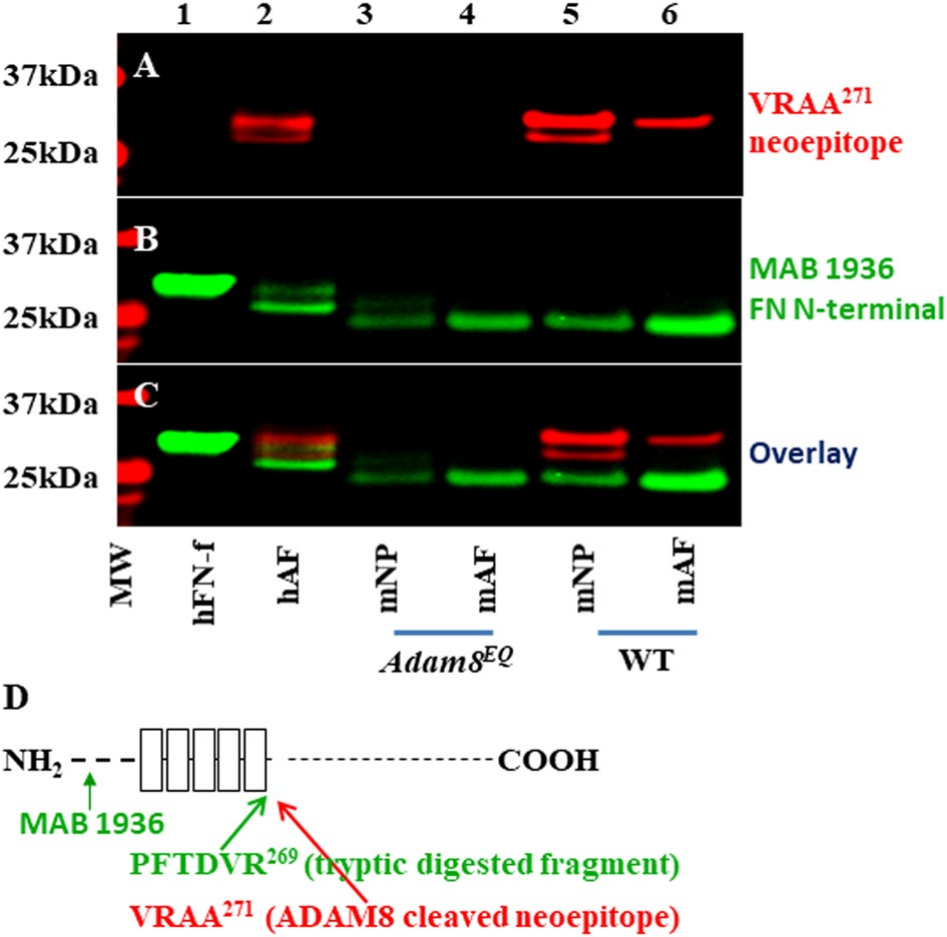
ADAM8 cleaves fibronectin, and *Adam8*^*EQ*^ mutation abrogates its enzymatic activity. *MW* molecular weight markers; Lane 1: human plasma FN-f (control); Lane 2: hAF, human annulus fibrosus (AF) tissue lysate (control); Lanes 3&4: mouse nucleus pulposus (mNP) & mAF from *Adam8*^*EQ*^ mice; Lanes 5&6: mNP and mAF tissues from wild type (WT) mice. (**A**) membrane probed with an antibody recognizing the FN neoepitope (VRAA; shown in red); (**B**) probed with monoclonal antibody (MAB) 1936 recognizing the FN N-terminal domain (shown in green); (**C**) panels (**A**,**B**) superimposed. (**D**) schematic drawing of the FN-f resulting from trypsin or ADAM8 digestion.

The *Adam8*^*EQ*^ mouse harbors a Glutamic Acid (E) to Glutamine (Q) mutation at amino acid 330 in the *Adam8* gene. NP and AF were dissected from WT and *Adam8*^*EQ*^ mice at 9 months of age. The neoepitope of fibronectin fragment (FN-f) resulting from ADAM8 cleavage (VRAA^271^) is shown in red, and the fibronectin N-terminal domain (recognized with antibody mAB1936), is shown in green (illustrated in Fig. 1D). Equal amounts of proteins per group were loaded on gels followed by Western blotting. Fibronectin neoepitope VRAA^271^ resulting from ADAM8 cleavage was absent in IVDs of *Adam8*^*EQ*^ mice, while it was present in WT mice, suggesting that ADAM8 is required for production of this neoepitope (n = 4; lanes 3&4 and 5&6; Fig. 1A). There were minor FN fragments containing the VRAA^271^ neoepitope, likely resulting from partial degradation (panel A, lane 5). Human plasma FN-f resulting from tryptic digest in the first lane was loaded as negative control because it is 2 amino acids shorter than the fragment containing neoepitope VRAA^271^^31^, and was therefore not recognized by the neoepitope antibody (panel B, lane 1). Samples of human AF tissue, known to contain the neoepitope VRAA^271^, did show a positive band recognized by the neoepitope antibody (red), and also contained fragments recognized by the mAB1936 (shown in green, lane 2, panels A&B). These results clearly show that the *Adam8*^*EQ*^ mutation effectively inactivates ADAM8 proteolytic activity directed against fibronectin in mouse IVDs. Superimposition of panels A and B showed that there are slightly smaller FN N-terminal fragments recognized by mAB1936. These fragments are present in both human and mouse samples including samples from mice with *Adam8*^*EQ*^ mutation (Panels B&C, lanes 1–6), suggesting that there may be enzymes other than ADAM8 capable of cleaving FN.

### *Adam8* gene expression in intervertebral discs (IVDs) of Adam8^EQ^ and WT mice

**Figure 2:**
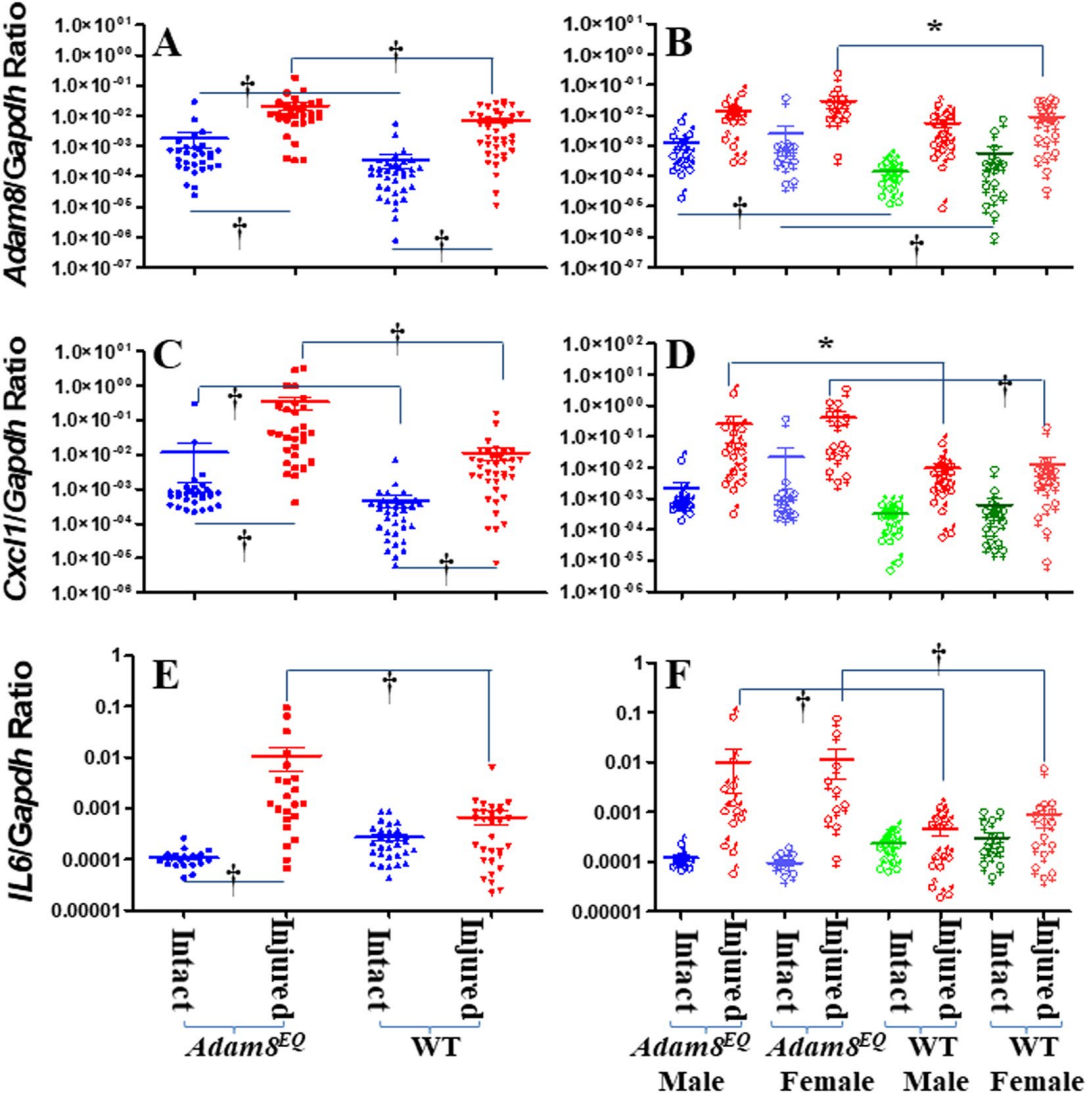
*Adam8* and Pro-inflammatory Gene Expression in the Injured Mouse Tail Intervertebral Disc. (**A**,**B**) *Adam8* gene expression; (**C**,**D**) *Cxcl1* gene expression; (**E**,**F**) *IL6* gene expression. *WT* wild type. Each point shows data from one intervertebral disc from an individual mouse. ^**†**^p ≤ 0.01; *p ≤ 0.05.

Since the *Adam8*^*EQ*^ mutation is a one nucleotide substitution, the *Adam8* gene expression remains under the control of the endogenous promoter in *Adam8*^*EQ*^ mice. Here we examined *Adam8* gene expression levels in these mice, compared with WT mice on DBA background. Consistent with previous findings^16,17^, *Adam8* gene expression was elevated in injured IVDs compared with that in adjacent intact discs in WT mice (n = 38 mice, p < 0.0001). In *Adam8*^*EQ*^ mice, *Adam8* gene expression was also elevated in injured discs compared with controls (n = 30 mice, p < 0.0001). Surprisingly, *Adam8* gene expression was higher in both injured and intact discs in *Adam8*^*EQ*^ mice than WT mice (n = 30 and 38 mice/group respectively, p ≤ 0.0001; Fig. 2A).

A separate comparison of *Adam8* gene expression in male and female mice revealed that *Adam8* gene expression was higher in intact discs of *Adam8*^*EQ*^ mice than in WT mice of both sexes (p < 0.0100). In female mice, *Adam8* gene expression in injured discs of *Adam8*^*EQ*^ mice was higher than in WT mice (n = 14 and 19 mice, respectively; p = 0.0129). There were no statistically significant differences in *Adam8* gene expression between male and female mice in intact or injured discs in mice of either genotype (p > 0.0500; Fig. 2B).

### *Cxcl1* gene expression in IVDs of Adam8^EQ^ and WT mice

*Cxcl1* gene expression was higher in injured IVDs than adjacent intact controls, in both *Adam8*^*EQ*^ and WT mice (n = 30 and 38 mice/group respectively, p < 0.0001). *Cxcl1* gene expression was higher in both injured and intact IVDs of *Adam8*^*EQ*^ mice than in WT mice (p < 0.0100; Fig. 2C).

When mice of different sexes were compared separately, *Cxcl1* gene expression in injured discs was found to be higher in *Adam8*^*EQ*^ than WT mice of both sexes (p = 0.0121 in males; p < 0.0001 in females). There were no significant differences in *Cxcl1* gene expression between male and female mice, in intact or injured discs of *Adam8*^*EQ*^ and WT mice (p > 0.0500; Fig. 2D).

### *IL6 *gene expression in IVDs of Adam8^EQ^ mice and WT mice

At 1-week post needle puncture, *IL6* gene expression was elevated in injured IVDs compared with that in adjacent intact discs in *Adam8*^*EQ*^ mice (n = 22 mice, p < 0.0001). In WT mice, however, the difference in *IL6* gene expression between injured and intact discs was not statistically significant (n = 30 mice, p = 0.6623). In injured IVDs, *IL6* gene expression was higher in *Adam8*^*EQ*^ than in WT mice (n = 22 and 30, respectively; p < 0.0001), but the difference between intact discs in *Adam8*^*EQ*^ and WT mice was not statistically significant (p = 0.1411; Fig. 2E). When data from male and female mice were analyzed separately, *IL6* gene expression was higher in injured *Adam8*^*EQ*^ than WT mice of both sexes (p < 0.0100), but there was no statistically significant difference between intact discs in *Adam8*^*EQ*^ and WT mice of either sex (p > 0.0500; Fig. 2F).

### Col1 and Col2 gene expression in IVDs of Adam8^EQ^ mice and WT mice

**Figure 3:**
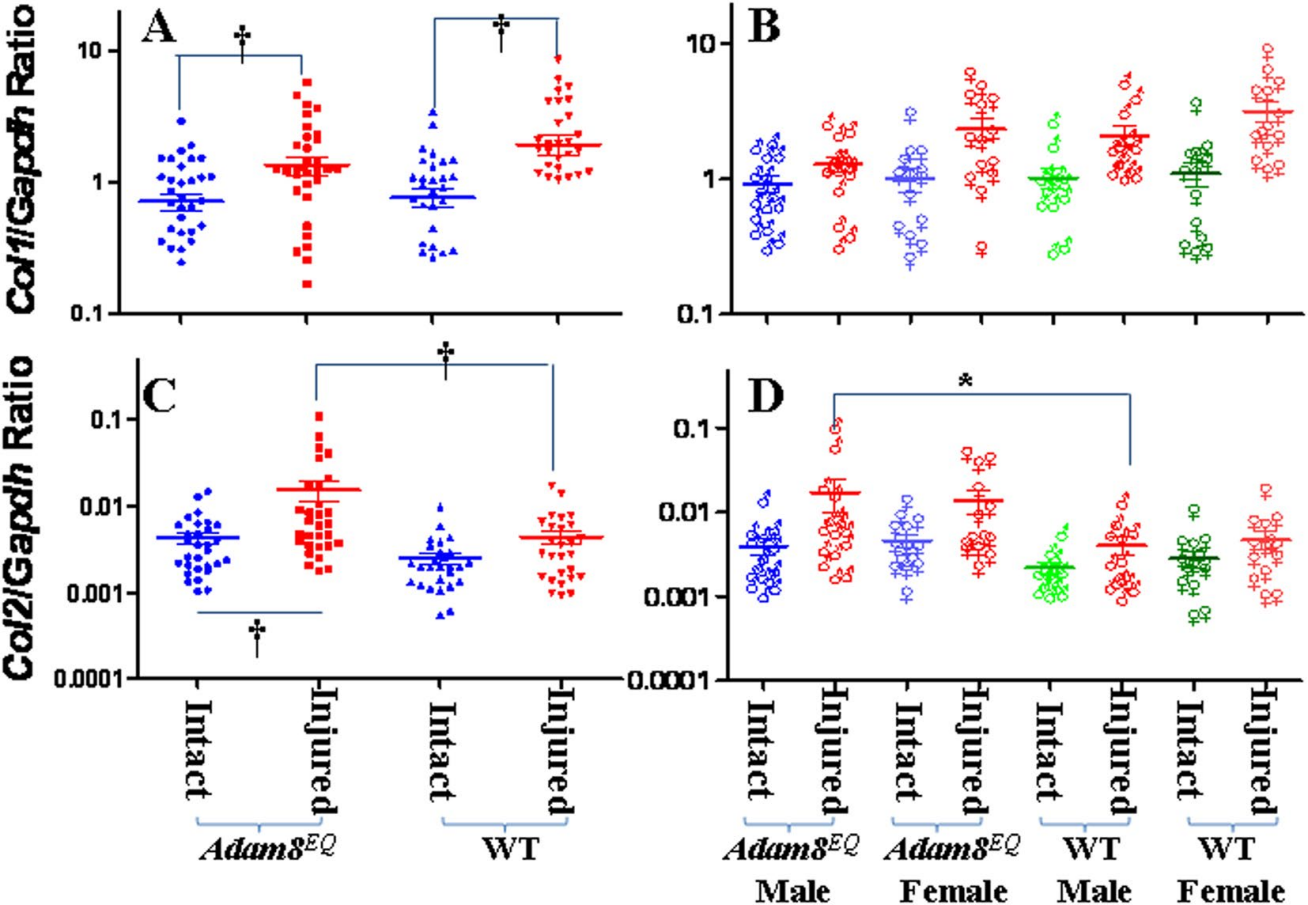
Collagen gene expression in the injured mouse tail intervertebral disc. (**A**,**B**) *Col1a1* gene expression; (**C**,**D**) *Col2a1* gene expression. *WT* wild type. Each point shows data from one intervertebral disc. ^**†**^p ≤ 0.01; *p ≤ 0.05.

Type I and Type II are major collagens in IVDs, and expression levels of these molecules have been shown to change in response to injury^16,17,27^. As expected, alpha chain of type I collagen (*Col1a1)* gene expression increased with injury, in both *Adam8*^*EQ*^ and WT mice (n = 30 and 27, p = 0.0029 and p < 0.0001, respectively). There was no difference in *Col1a1* gene expression in WT mice versus *Adam8*^*EQ*^ mice, either in injured or intact discs (p > 0.0500; Fig. 3A). A separate analysis of different sexes found no significant difference in *Col1a1* gene expression in male and female mice of either genotype (p > 0.0500; Fig. 3B).

Alpha chain of type II collagen (*Col2a1)* gene expression increased with injury in *Adam8*^*EQ*^ mice (n = 30, p = 0.0010). There was no significant increase in *Col2a1* gene expression in WT mice in response to injury (n = 27, p = 0.4052). Interestingly, *Col2a1* gene expression was higher in injured IVDs of *Adam8*^*EQ*^ than WT mice (p = 0.0006), but no significant difference was found between intact discs of *Adam8*^*EQ*^ and WT mice (Fig. 3C). When the animals of different sexes were analyzed separately, there was higher *Col2a1* gene expression in injured IVDs of male *Adam8*^*EQ*^ mice compared with WT male mice (n = 16 and 13, respectively; p = 0.0434). In injured discs of female mice, there was no significant difference in *Col2a1* gene expression between *Adam8*^*EQ*^ and WT mice (n = 14 mice/group; p = 0.1380). There was no significant difference between intact discs of male or female *Adam8*^*EQ*^ and WT mice (p > 0.0500; Fig. 3D).

### Tail IVD showed consistent histological changes following injury in Adam8^EQ^ mice and WT mice

**Figure 4:**
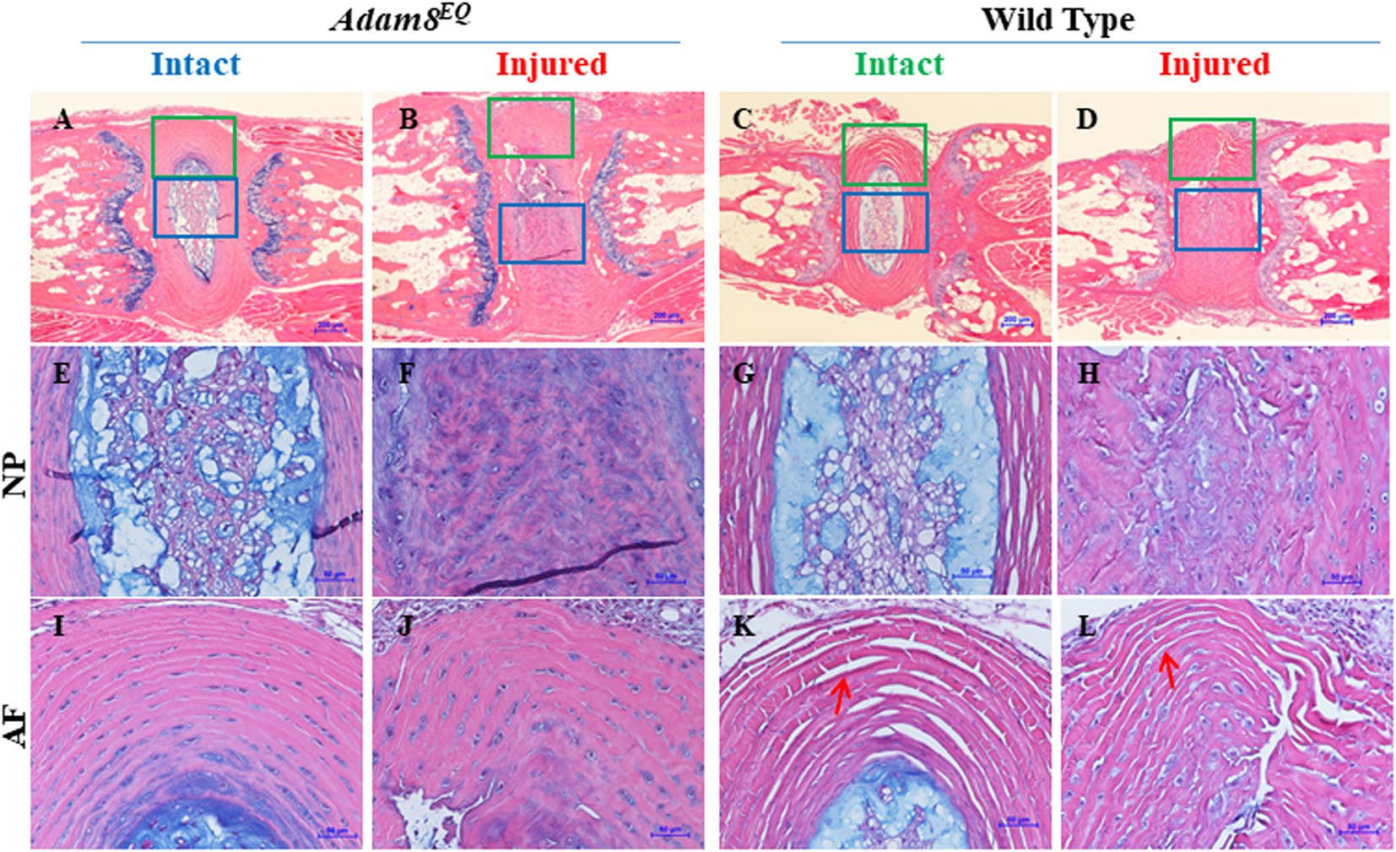
Alcian Blue staining demonstrating histologic changes including loss of proteoglycan in *Adam8*^*EQ*^ and wild type (WT) mice at 1 week after coccygeal intervertebral disc (IVD) injury. Intact and adjacent injured IVD were stained with Alcian blue; E through H and I through L are magnified images of blue and green outlined regions in **A**–**D**, respectively. *AF* annulus fibrosis, *NP* nucleus pulposus. Scale bars, 200 µm (**A**–**D**); 50 µm (**E**–**L**).

**Figure 5:**
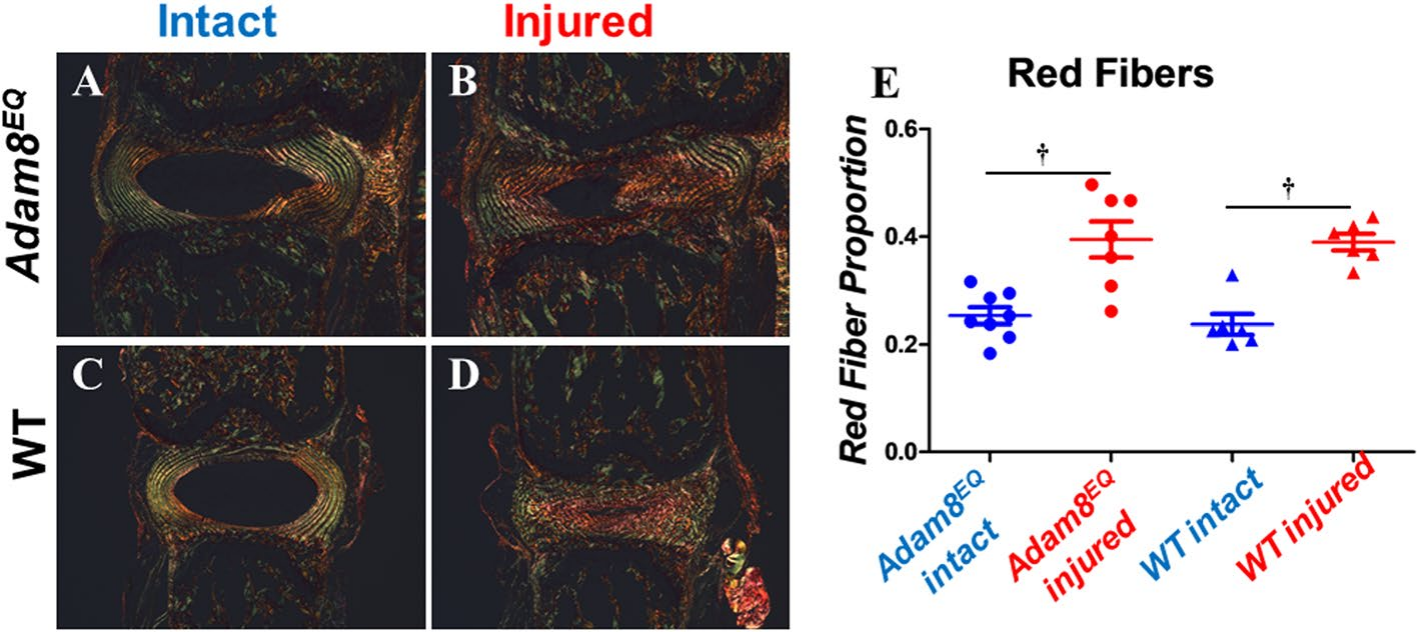
Red (thickest) fiber proportion increased in injured mouse intervertebral disc compared with intact controls in both *Adam8*^*EQ*^ and wild type (WT) mice. Left: injured and intact discs stained with picrosirius red (PSR) by polarized microscopy; right: red (thickest) fiber proportion; ^**†**^p < 0.01.

**Figure 6:**
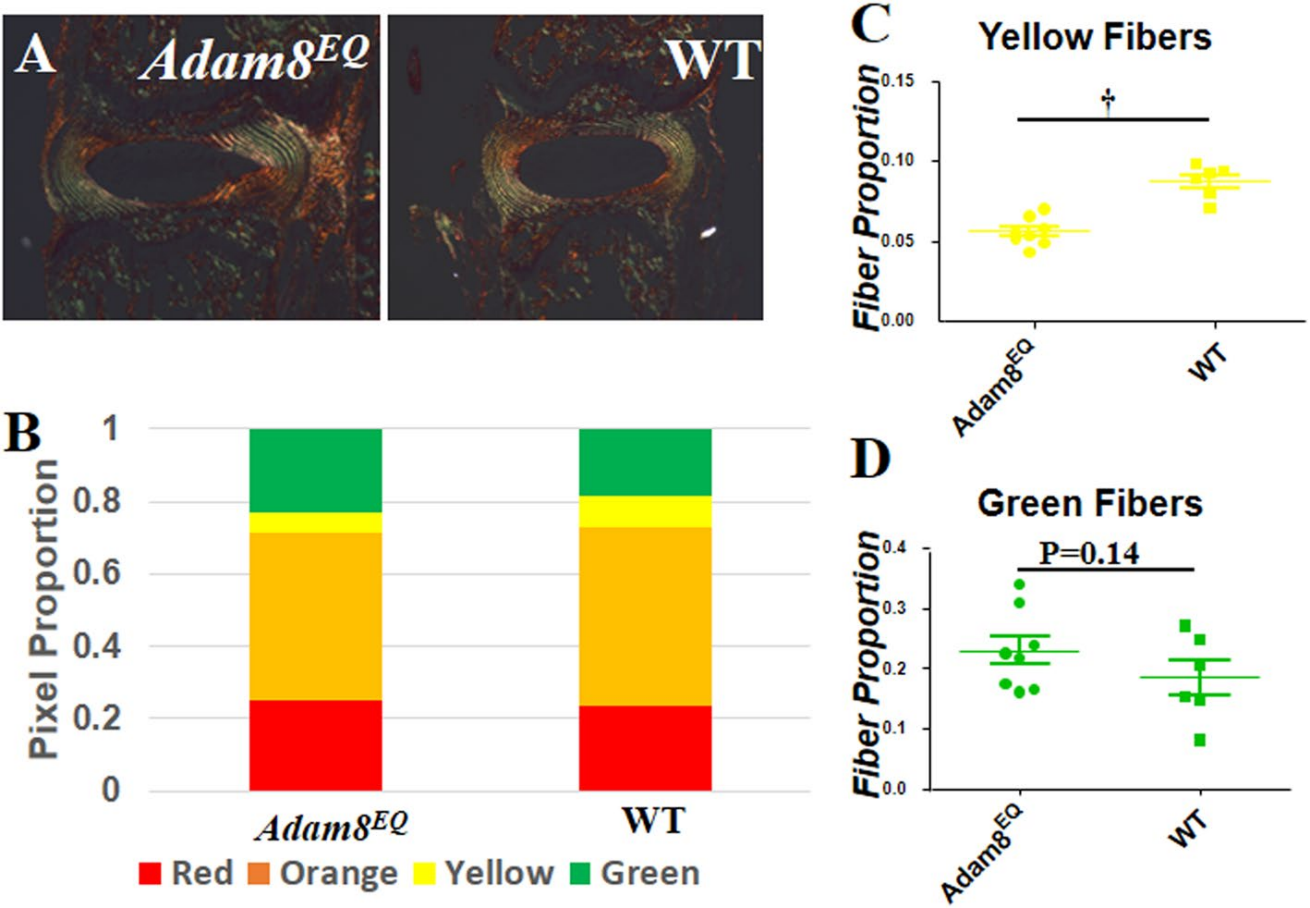
Fiber size differ between the intervertebral discs of *Adam8*^*EQ*^ and wild type (WT) mice. (**A**) discs stained with picrosirius red under polarized microscope; (**B**) pixel proportion of thin (green), intermediate (yellow and orange), and thick (red) collagen fibers; (**C**,**D**) yellow and green fiber pixel proportion in *Adam8*^*EQ*^ and WT mouse discs; ^**†**^p < 0.01.

We have examined histological features of mouse tails following injury, by staining mid-sagittal sections with Alcian Blue to reveal proteoglycans (Fig. 4), and picrosirius red (PSR) to reveal collagen fibers (Figs. 5, 6). In both *Adam8*^*EQ*^ mice and WT mice, there were consistent histological changes in both male and female IVDs following injury: IVD tissues showed loss of normal NP architecture, disruption of AF, and loss of proteoglycan in both *Adam8*^*EQ*^ and WT mice (n = 8 mice/group; Fig. 4). Specifically, normal NP and AF architecture and proteoglycan were revealed by Alcian Blue staining and H&E counter staining, in intact *Adam8*^*EQ*^ mice (Fig. 4A,E,I) and WT mice (Fig. 4C,G,K). Following injury, IVD tissues showed loss of normal IVD architecture, and loss of proteoglycan in *Adam8*^*EQ*^ mice (Fig. 4B,F,J). Normal NP architecture was lost (Fig. 4F), and AF ring distortion and interruption occurred (Fig. 4J). Similar changes occurred in WT mice following disc injury (Fig. 4D,H,L). Anecdotally, a ‘processing artifact’ typified by separation of annular rings appeared to be more frequent in WT mice (arrows in Fig. 4K,L) than in *Adam8*^*EQ*^ mice (Fig. 4I,J). This difference coincided with higher *Col2a1* expression in discs of *Adam8*^*EQ*^ mice than WT mice. It is intriguing to ask if and how the two observations are connected. No significant differences in cartilaginous endplate were observed in *Adam8*^*EQ*^ or WT mice.

### Increased proportion of thick (red) collagen fibers in both Adam8^EQ^ and WT mice following tail IVD injuries

It is known that the color of collagen fibers (or fiber bundles) stained with PSR and viewed under circularly polarized light correlates with collagen thickness; as fiber thickness increases, the color changes progressively from green to yellow, orange, and red^32–34^. Quantification of PSR-stained collagen fibers revealed that following injury, red (thick) fibers were elevated in both *Adam8*^*EQ*^ (n = 7 mice, p = 0.0003) and WT mice (n = 6 mice, p = 0.0001; Fig. 5). Enrichment in thick collagen fibers suggests formation of collagen fibers by cells occupying the NP space after injury. The inner AF tissues have been shown to encroach on the NP space after injury^35^. The data shown here suggest that these tissues, containing degenerative NP and encroaching inner AF, contained a larger proportion of thick collagen fibers than intact IVD tissues. There were no significant differences when comparing red fiber proportion in intact or injured discs of the *Adam8*^*EQ*^ and WT mice (p > 0.0500). Yellow (intermediate thickness) fiber proportion decreased following injury, both in *Adam8*^*EQ*^ mice (intact: 0.0562; injured: 0.0441; p = 0.0264) and WT control mice (intact: 0.0876; injured: 0.0457; p < 0.0001). Differences in green (thin) fiber proportion in injured and intact discs were not statistically significant. In the *Adam8*^*EQ*^ mice, green fiber proportion in intact and injured discs was 0.2301 ± 0.0182 and 0.1752 ± 0.0207 (n = 8 and 7, respectively; p = 0.0717). Similarly, in WT mice, green fiber proportion in the intact and injured discs was 0.1854 ± 0.0210 and 0.1375 ± 0.0210 (n = 6 mice/group, p = 0.1509).

### Lower proportion of intermediate (yellow) collagen fibers in Adam8^EQ^ than WT mice

In intact IVDs, *Adam8*^*EQ*^ AFs contained lower proportion of yellow (intermediate) fibers than those in WT mice (n = 8 and 6 mice, respectively; p < 0.0001; Fig. 6C). Relative proportion of green fibers showed a substantial inter-mouse variation; therefore, the differences between the *Adam8*^*EQ*^ and WT mouse discs were not statistically significant (p = 0.1364; Fig. 6D). A decrease in yellow fiber proportion in *Adam8*^*EQ*^ mice suggests that accumulation of the intermediate sized collagen fibers in AF of mutant mice was affected by ADAM8 inactivation, likely due to alterations of assembly and orientation of collagen fibrils. This difference may result in altered resistance to mechanical forces, and may account for reduced frequency of fissures in *Adam8*^*EQ*^ AF (compared with WT) during tissue dissection and processing.

## Discussion

Previously, we have identified ADAM8 as a fibronectin-cleaving enzyme in human degenerative IVDs, thus establishing its clinical relevance in disc degeneration^3^. The present study with *Adam8* mutant mice aims to elucidate molecular mechanisms in ADAM8-mediated disc disease. We used a mouse line (*Adam8*^*EQ*^) with a single nucleotide substitution, resulting in replacement of the Glutamic acid (E) at position 330 with a Glutamine (Q). This single amino acid substitution in ADAM8 proteolytic domain prevents prodomain removal required for ADAM8 activation^13^. *Adam8*^*EQ*^ mice develop normally, with similar body weight and litter size to those of WT mice. We have shown here that the FN-f with VRAA neoepitope is absent in *Adam8*^*EQ*^ mouse NP and AF but present in WT mouse tissues, confirming that the mutation effectively abrogated ADAM8 enzymatic function. On the Western blot, human plasma FN-f resulting from trypsin digestion was not recognized by the antibody to the VRAA neoepitope (Fig. 1), indicating that this antibody is highly specific. There are at least 2 bands with MW between 25 and 37 kD that are recognized by this antibody, likely resulting from proteolysis at the N-terminus. Additionally, there are FN-fs not recognized by the VRAA neoepitope antibody in both human disc tissue and mouse tissues from *Adam8*^*EQ*^ and WT mice, which may be products resulting from the action of other enzymes such as metalloproteinases (MMPs)^36^ and high temperature requirement serine protease A1 (HTRA1)^37^, both known to cleave fibronectin and generate fragments near the N-terminus.

We challenged mouse *Adam8*^*EQ*^ IVD with a needle puncture. The puncture causes a partial disc herniation, since we frequently saw gelatinous material at the needle tip when the needle was withdrawn. To visualize the degenerative disc, we have used a Col2CreER/tdTomato system which labeled the inner AF red^38^. After injury, fibrocartilage in the degenerative IVDs consisted of residual diseased NP cells and encroaching inner AF cells^35^. Gene expression in *Adam8*^*EQ*^ mice was compared with that in WT mice on the same genetic background since genetic background was shown to affect inflammatory cytokine and collagen gene expression^16^. *Adam8* gene expression in injured *Adam8*^*EQ*^ mouse IVDs was elevated compared with that in WT mice, likely due to compensatory mechanism(s) reflecting absence of ADAM8 activity. To our surprise, *Cxcl1* gene expression was elevated in both intact and injured *Adam8*^*EQ*^ mouse discs compared with WT mouse discs. *IL6* gene expression in injured *Adam8*^*EQ*^ mouse discs was also higher than in WT mice. One limitation is that only gene expression has been examined in this study. In the future, protein distribution and level could be compared in *Adam8*^*EQ*^ and WT mice by immunostaining with histomorphometric quantification. It is unclear what roles cytokines play in the maintenance of intact discs and in repair of injured discs. Zack et al. found decreased inflammation in knee joints of *Adam8*^*EQ*^ mice compared with those of WT mice, in response to collagen- and LPS-induced inflammation^24^. These investigators used a model of joint inflammation induced by a combination of collagen and LPS^24^, which likely induced autoantibodies to various collagen epitopes, as well as activating the innate immune system with LPS. In the tail IVD injury model, there is loss of intradiscal pressure immediately following injury, and subsequent NP cell dissociation^35^ with loss of N-cadherin^35,39^. These findings highlight the complexities of inflammation pathways, both in synovial joints and IVDs. Future work is needed to clarify how injury induces expression of *Adam8* and other inflammatory genes and what roles inflammation has in disc repair.

Type II collagen (*Col2a1*) gene expression was higher in injured *Adam8*^*EQ*^ IVDs, compared with injured WT discs. Type II collagen is important for the biomechanical properties of NP and inner AF, and increased production of this collagen may improve the function of IVD. Therefore, elevated *Col2a1* gene expression is encouraging if inhibiting ADAM8 enzymatic activity is considered a candidate therapeutic strategy. Histological examination did reveal that AF of the *Adam8*^*EQ*^ mouse IVDs have fewer fissures between annular rings than AF of WT mice. The fissures between annular rings are artifacts resulting from tissue preparation, and may also reflect the extracellular matrix content and organization of the tissue. We have detected differences in fiber size between injured and intact discs, in both ADAM8-mutant and WT mice. Yellow (intermediate size) fibers differed between intact discs in *Adam8*^*EQ*^ and WT mice. Green (small) fibers in mice of the two genotypes did not differ significantly, and a larger number of mice will be examined in the future in an effort to confirm this finding. The method used here quantified the hue of PSR stained tissue sections under polarized light, which correlates with, but does not directly measure, the diameter of collagen fibers (or fiber bundles)^32–34^. Scanning electron microscopy^40,41^ and single harmonic generation imaging^42^ methods are the gold standards in measuring collagen fiber size, and should therefore be explored in the future. In addition, immunostaining of collagen and aggrecan degradation products, and biomechanical testing would help to further establish whether the IVDs of *Adam8*^*EQ*^ mice have improved structure and biomechanical properties than those of WT mice. The relatively minor impact of ADAM8 inactivation on disc morphology in young adult mice may suggest that ADAM8 has a small role in IVD development, but the “small” effects of ADAM8 may be cumulative and clinically significant over a long time-span. Examination of older mice is an important future direction.

The tissue changes during the proposed 1-week time frame reflect inflammation and repair in response to an acute injury, and longer time after injury will reflect tissue remodeling and chronic degeneration (as a future direction). Because of similarities in histological features and gene expression at the 1- and 2-week time points, we are planning to conduct sample collections at multiple time points such as: 1–2 days, 1 week and 4 weeks post injury. Such comprehensive analysis for *Adam8*^*EQ*^ mice comparing with WT controls will shed light on events at immediate, acute, and chronic phases post injury. Another limitation of the current study is that only a small number of genes have been examined. RNAseq assay will be performed in the future to explore differences between injured/intact and mutant/WT discs, to yield unbiased biomarkers and broaden the scope of our studies. Future work will also include histological grading of degenerating discs, with special attention paid to endplate, bone and soft tissues adjacent to the injured IVD.

In summary, we previously identified ADAM8 as the fibronectin-cleaving enzyme in human IVD tissues, and found also that the active form of this enzyme correlated with increased degree of IVD degeneration. In the current study, we have examined a mouse line with ADAM8 proteolytic function inactivated (*Adam8*^*EQ*^). The ADAM8 inactivation abrogated the generation of a fibronectin cleavage product bearing the neoepitope VRAA. In response to injury, *Cxcl1*, *IL6*, and *Col2* gene expression was higher in discs of *Adam8*^*EQ*^ than WT mice. Histological differences, including collagen fiber sizes, are very subtle and will require further analysis. The subtle phenotype of *Adam8*^*EQ*^ mice may be due, at least in part, to multiple enzymes (e.g., MMPs, HTRA1) that cleave fibronectin. Since degeneration of the IVDs develops over decades in humans, the small effects of ADAM8 may be biologically significant. Future examination of additional time points post injury, and aging *Adam8*^*EQ*^ mice might further shed light on disc inflammation, pain, and degeneration. This mechanistic approach may tease out contributing factors and therapeutic targets for IVD degeneration and related back pain, and ultimately benefit patients.

## Materials and methods

### Mice

All animal experimental procedures were approved by the Institutional Animal Care and Use Committee of the University of Pennsylvania or Rush University Medical Center, and all methods were performed in accordance with the relevant guidelines and regulations. The study was carried out in compliance with the Animal Research: Reporting of In Vivo Experiments (ARRIVE) guidelines. A breeding pair of *Adam8*^*EQ*^ mice was transported from Rush University Medical Center to the University of Pennsylvania (a generous gift from Dr. AnneMarie Malfait). A breeding pair of DBA/1LacJ mice (the Jackson Laboratory, Bar Harbor, ME, USA) was used to produce the same strain of wild type (WT) mice as controls. Mice were housed under pathogen-free conditions with environmental enrichment, with up to 5 mice per cage. A total of 76 mice were used in this study. For the examination of ADAM8 proteolytic activity by assessing fibronectin (FN) fragments, 4 *Adam8*^*EQ*^ and 4 DBA mice (as WT controls) at 9 months of age were used. For tail IVD injury studies, 30 *Adam8*^*EQ*^ and 38 WT mice, age 10–12 weeks at the time of surgery, were used.

### NP and AF dissection, protein isolation, and Western blot analysis

The lumbar and coccygeal vertebrae were isolated under a dissecting microscope (VistaVision, VWR International, Radnor, PA). Lumbar and coccygeal NP and AF tissues were pooled for each animal. Specifically, the gelatinous NP was scraped off with a scalpel^39^. AF tissues, identified by their concentric rings, were shaved off the cartilaginous endplate with a scalpel. For Western blotting, the NP and AF tissues were snap frozen in liquid nitrogen and stored at − 80 °C until use. For protein extraction, frozen tissues were crushed into a fine powder using a pre-cooled Bio-Pulverizer (Biospec Products, Bartlesville, OK). The protein from the powdered tissue was extracted with lysis buffer (Cell Signaling, Danvers, MA) containing protease inhibitors (Roche, Basel, Switzerland) at 4 °C for 24 h. Protein concentration was determined using a Pierce BCA protein assay kit (Thermo Fisher Scientific Inc., Rockford, IL). For Western blots, 7 μg of protein extracts from disc tissues were treated with 0.1 U/ml chondroitinase ABC (Sigma) in 50 mM Tris–acetate-EDTA buffer at 37 °C for one hour. A human degenerative AF tissue, Pfirrmann grade IV^43^, which was known to contain fibronectin fragments (FN-fs)^3^, served as a positive control. A commercially available 29 kDa human FN N-terminal fragment (hFN-f, resulting from digestion of plasma fibronectin with Cathepsin D and Trypsin, Sigma, St. Louis, MO) was also used as a positive control. To identify FN-fs containing the N-terminus fragment and the neoepitope VRAA271, the following primary antibodies were used: mouse monoclonal antibody (mAB) to the 29 kDa FN N-terminal fragment (mAB1936, Chemicon/Millipore Clone 616, Temecula, CA) and rabbit polyclonal neoepitope antibody VRAA271 (kindly supplied by Pfizer, Inc.)^18^. Specifically, proteins were resolved on NuPAGE 4–12% Bis–Tris Gels (Invitrogen Life Technologies, Carlsbad, CA), and were then transferred on to Immobilon Membrane, PVDF type (Millipore, Burlington, MA). Membranes were incubated with mAB1936, followed by infrared (IR) Dye 680-conjugated goat anti-mouse IgG. The membrane was then incubated with the rabbit polyclonal neoepitope antibody VRAA271, followed by IRDye 800-conjugated goat anti-rabbit IgG (diluted 1:20,000; LI-COR Biosciences, Lincoln, Nebraska, USA). An infrared imager (LI-COR Odyssey, LI-COR Biosciences) was utilized to allow identification of two antigen epitopes simultaneously. The experiment was repeated, with 4 NP and AF tissues from 4 different *Adam8*^*EQ*^ and WT mice. Membranes were analyzed using Odyssey Infrared Imaging System software (LI-COR Biosciences). Full length Western blotting images have been included as supplemental material (Fig. S1).

### Tail injury surgery

Surgery was performed as described previously^17^. Specifically, each mouse was anesthetized with Ketamine (90 mg/kg) and Xylazine (10 mg/kg) subcutaneously. Under anesthesia, the skin was cleaned with betadine. Under fluoroscopic guidance with a mini C-arm (OrthoScan FD Pulse Mini C-Arm, Orthoscan Inc., Scottsdale, AZ), the mouse coccygeal (Co) IVDs were identified, and a 26G needle was inserted into the IVD space until the needle tip reached approximately 2/3 of the disc thickness. Care was taken not to puncture the opposing AF. Indeed, when the opposing AF wall was damaged, a more severe injury was seen on MRI^25^. In the current study, the Co3/4 and Co5/6 IVDs in each mouse were injured, while Co4/5 and Co6/7 served as intact controls. The animals behaved normally (by observing breathing, eating, ambulation, etc.), and did not require medication for pain; no adverse event such as infection or bleeding was noted following surgery. Mice were euthanized by exposure to CO_2_ at one week after tail disc injury. From each mouse tail, Co3/4 (injured) and Co4/5 (intact control) discs were isolated individually for RNA extraction. Co5/6 (injured) and Co6/7 (intact control) were isolated en bloc for histological examination.

### RNA isolation and quantitative real-time PCR

NP and AF were analyzed together, since it is not feasible to separate NP from AF in the injured IVDs^35^. Specifically, the IVDs were separated from their adjacent cartilaginous endplates and bone with a scalpel, under a dissecting microscope (VistaVision, VWR International, Radnor, PA). Total cellular RNA was isolated by the Trizol method. The isolated IVD tissues were soaked in RNALater (Ambion, Foster City, CA) overnight, and stored at − 80 °C until extraction. On the day of RNA extraction, RNALater was removed and the tissues were snap frozen with liquid nitrogen, and then transferred into Trizol (Invitrogen, Carlsbad, CA). The tissues were homogenized with a homogenizer with disposable OmniTip probes for hard tissue (Omni International, Kennesaw, GA). RNA was precipitated with 70% ethanol, and was further purified using a RNeasy Micro Kit (Qiagen), as described previously^17^. RNA concentration was determined using a Synergy H4 Hybrid Reader (BioTek, Winooski, VT, USA). To generate cDNA, all RNA from each IVD (7–20 ng/µl, 50 µl total volume per sample) was used as template in a reverse transcriptase reaction using the SuperScript VILO cDNA synthesis kit (Life Technologies, Carlsbad, CA, USA) containing random hexamers and added polyDT primers (Invitrogen, Carlsbad, CA, USA). Sequences of cDNA were retrieved from Ensembl (release 84, March 2016). Primers for real-time PCR were designed using Primer-BLAST^44^, and synthesized by Invitrogen (Carlsbad, CA, USA). For each PCR reaction, cDNA, SYBR Select master mix (Life Technologies, Carlsbad, CA, USA), and primers (working concentration 0.5 μM) were mixed, and deionized water was added to a total volume of 20 μl per reaction. MicroAmp Optical 96-well reaction plates (Applied Biosystems, Foster City, CA) with 20 µl of reaction mixture/well were sealed with optical adhesive film (Life Technologies, Frederick, MD) and run in a ViiA7 real-time PCR system (Applied Biosystems, Foster City, CA) using the following program: (1) 50 °C for 2 min, (2) 95 °C for 2 min, (3) 95 °C for 15 s, (4) 58 °C for 1 min, (5) repeat steps 3 and 4 a total of 40 times. Single products were confirmed by determining melting curves at the conclusion of the reaction. Each individual gene expression to *Gapdh* ratio was calculated (equal to 2^−ΔCt^)^45^.

### Histological evaluation

The IVDs and portions of the adjacent bony vertebral bodies were isolated immediately after euthanasia, and fixed with 4% paraformaldehyde for 24 h. The bone-disc-bone segments were decalcified with a solution consisting of 12.5% EDTA for approximately one week, with shaking, until the bony portion was completely decalcified. The tissues were then dehydrated and embedded in paraffin and sectioned to a 5 µM thickness. The sections were subsequently stained with Hematoxylin and Eosin (H&E), Alcian Blue (to reveal proteoglycan) and PSR (collagen fiber). For Alcian Blue staining, the tissue sections were stained with 1% Alcian Blue Solution (Poly Scientific R&D Corp., Bay Shore, N.Y.) for 5 min, followed by Hematoxylin for 5 min and Eosin for approximately 20 s. For PSR staining, sections were stained with 0.1% PSR (Sigma) for 45 min. All samples were examined under a light microscope (Nikon) and photographed. Representative sections from each group were chosen for presentation.

### Quantification of collagen fiber size

The tissue sections, stained with PSR, were examined under a BXZ-700 microscope (Keyence, Itasca, IL, USA) with a circular polarizer filter and analyzed by ImageJ software (NIH Image). Briefly, the AF regions were cropped and converted to HSB Stack. We used the following hue definitions: red 2–9 and 230–256, orange 10–38, yellow 39–51, green 52–128^34^. The pixel numbers of red, orange, yellow, and green (the colors of collagen fibers in order of decreasing thickness) were calculated, and a histogram of the hue slice image was obtained and analyzed.

### Statistics

The differences in genes of interest (*Adam8*, *Cxcl1*, *Col1a1* and *Col2a1*) and house-keeping gene (*Gapdh*) cycle thresholds (ΔCT) were calculated for each injured/intact pair. Fold changes of each gene were calculated based on ΔCT. To assess differences in ΔCT between injured and intact tissues, a 3-factor analysis of variance (ANOVA) in repeated measures was used, where genotype and sex were grouping factors and injured/intact was the repeated measure. To adjust for multiple comparisons, post-hoc pairwise t-tests using the Tukey–Kramer method were performed for injured/intact differences within genetic background and sex. A p-value of < 0.05 was considered statistically significant. All analyses were performed using SAS statistical software (Version 9.4, SAS Institute, Cary, NC)^46^.

### Ethical approval statement

All animal use in this study was reviewed and approved by the Institutional Animal Care and Use Committee (IACUC) at the University of Pennsylvania, Philadelphia, PA. All authors approved the final version of the manuscript submitted. None of the authors have any professional or financial affiliations that may be perceived to have biased the presentation.

## Supplementary Information

Supplementary Legend.

Supplementary Figure S1.

## Abbreviations

ADAM8: A Disintegrin and metalloproteinase domain-containing protein 8
IL: Interleukin
CXCL1: C-X-C motif ligand-1
IVD: Intervertebral disc
