# Supplementary material for: Elevated inflammatory gene expression in intervertebral disc tissues in mice with ADAM8 inactivated
Source: Sci Rep. 2021 Jan 19;11:1804. doi: 10.1038/s41598-021-81495-y (PMC7815795; doi:10.1038/s41598-021-81495-y)
Supplement: Supplementary file 1 — Supplementary Legend. [file 41598_2021_81495_MOESM1_ESM.docx]

**Figure S1. ADAM8 cleaves fibronectin (FN), and *Adam8^EQ^* mutation abrogates its enzymatic activity. Panel A**: membrane probed with monoclonal antibody (MAB) 1936 recognizing the FN N-terminal domain (shown in green); **B**: the same membrane shown in panel A was further probed with an antibody recognizing the FN neoepitope (VRAA; shown in red). The bands in lanes 3-6 with apparent molecular weight of 50kDa, and multiple bands in lane 2 diminished with washing, after incubation with the FN neoepitope VARR and secondary antibody. These bands were presumed to be “nonspecific”. **hFN-f,** human plasma FN-f (control); **hAF,** human annulus fibrosus (AF) tissue lysate (Pfirrmann grade V, as positive control); **mNP and mAF:** mouse nucleus pulposus or AF tissues; ***Adam8^EQ^***: mutant mice; **WT:** wild type mice.
