# Supplementary figures and images for: Elevated inflammatory gene expression in intervertebral disc tissues in mice with ADAM8 inactivated
Source: Sci Rep. 2021 Jan 19;11:1804. doi: 10.1038/s41598-021-81495-y (PMC7815795; doi:10.1038/s41598-021-81495-y)

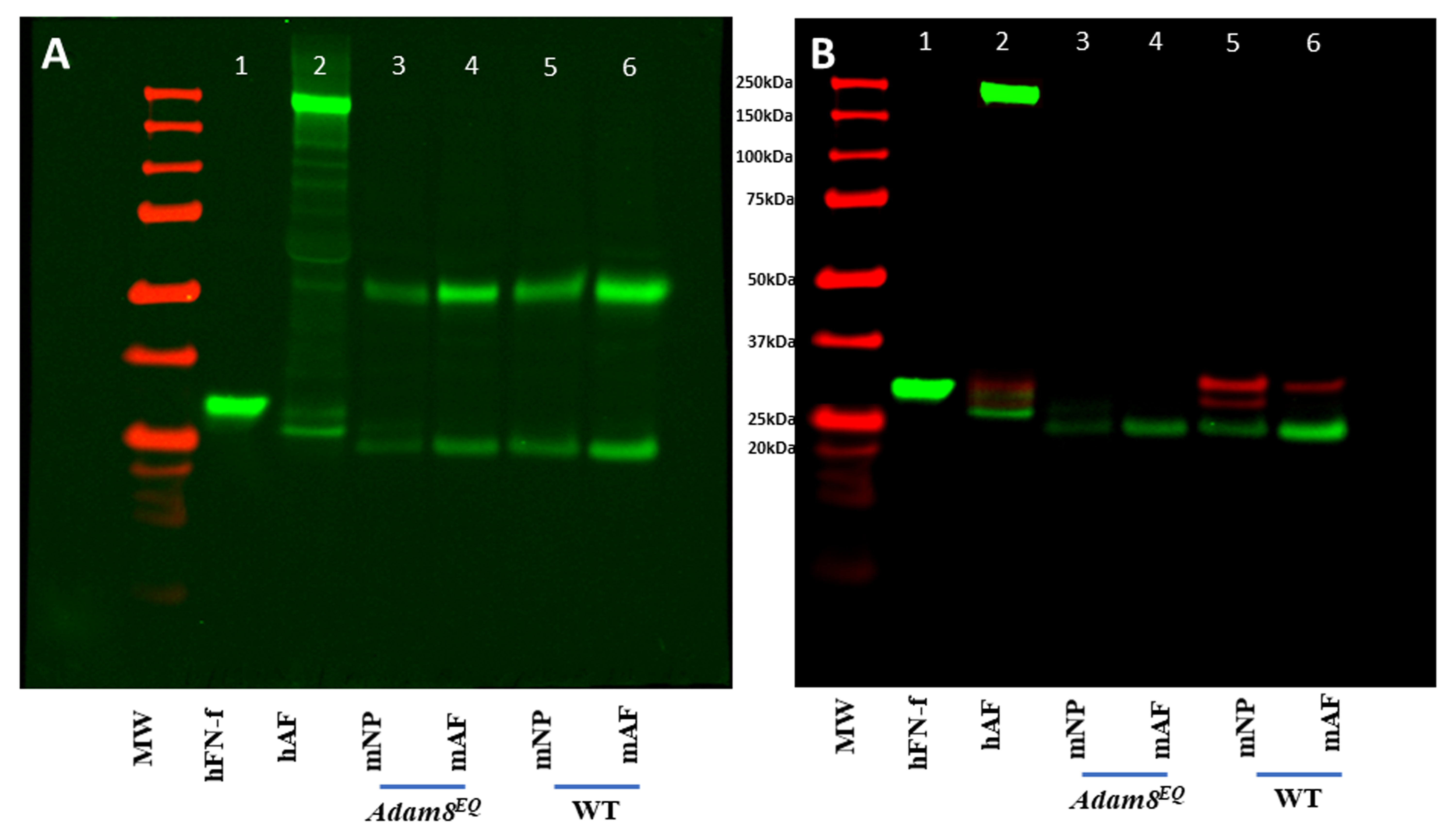

Supplement: Supplementary file 2 — Supplementary Figure S1. [file 41598_2021_81495_MOESM2_ESM.tif]
